# Effects of Different Types of Additional Fertilizers on Root-associated Microbes of Napa Cabbage Grown in an Andosol Field in Japan

**DOI:** 10.1264/jsme2.ME22013

**Published:** 2022-05-31

**Authors:** Seishi Ikeda, Kazuyuki Okazaki, Hirohito Tsurumaru, Takanori Suzuki, Masayuki Hirafuji

**Affiliations:** 1 Memuro Research Station, Hokkaido Agricultural Research Center, National Agriculture and Food Research Organization, 9–4 Shinsei-minami, Memuro, Kasai-gun, Hokkaido 082–0081, Japan; 2 Faculty of Agriculture, Kagoshima University, 1–21–24, Korimoto, Kagoshima 890–0065, Japan; 3 SUN AGRO Co., Ltd., 1470 Shiraoka, Shiraoka, Saitama 349–0218, Japan; 4 The University of Tokyo, 1–1–1, Midoricho, Nishi-Tokyo, Tokyo, 183–0002, Japan

**Keywords:** Additional fertilization, *Bipolaris*, Microbial community ana­lysis, Napa cabbage root, *Olpidium*

## Abstract

The effects of different types of additional fertilizations (a compound fertilizer and Chiyoda-kasei) on the root-associated microbes of napa cabbage grown in an Andosol field were investigated by molecular community ana­lyses. Most of the closest known species of the bacterial sequences whose relative abundance significantly differed among fertilizers were sensitive to nitrogen fertilization and/or related to the geochemical cycles of nitrogen. The fungal community on the roots of napa cabbage was dominated by two genera, *Bipolaris* and *Olpidium*. The relative abundance of these two genera was affected by the types of fertilizers to some extent and showed a strong negative correlation.

An understanding of the diversity and functionality of environmental microbes is a key factor for constructing a sustainable agricultural system as a green technology (Bulgari *et al.*, 2019). However, the practical utilization of the functionality of beneficial microbes remains challenging (Raymaekers *et al.*, 2020). One of the main reasons for this is most likely due to the lack of sufficient information on the interactions between beneficial microbes and diverse environmental factors, including fertilizers and pesticides (Ikeda *et al.*, 2010a). The impact of basal and additional fertilizations on plant-associated microbes has been assessed in the last decade (Ikeda *et al.*, 2010b, 2011, 2015; Rodríguez-Blanco *et al.*, 2015; Beltran-Garcia *et al.*, 2021; Wang *et al.*, 2022); however, the effects of qualitative differences in additional fertilizations remain unknown.

In contrast to a basal fertilizer, which is thoroughly mixed into soil at the beginning of cultivation, an additional fertilizer is often applied to the soil surface (top dressing fertilization). Chiyoda-kasei (SUN AGRO) is a unique fertilizer that is expected to have high solubility and rapid diffusibility from the surface of a field into soil, which is most likely due to its porous structure (Fig. S1A). By reacting liquid and gas in its production processes, each of the chemical components in a particle of Chiyoda-kasei is more evenly distributed within its particles than in a general compound fertilizer (Fig. S1A and B). These unique features of Chiyoda-kasei are advantageous for top dressing with an additional fertilization because they support the fast growth of crops with a rapid and balanced nutrient supply to soil. Therefore, Chiyoda-kasei may also have a unique impact on plant-associated microbes over a conventional compound fertilizer. The present study investigated the root-associated microbes of napa cabbage after top dressing with an additional fertilizer using Chiyoda-kasei and a conventional compound fertilizer.

The seeds of the napa cabbage (*Brassica rapa* var. *pekinensis*) cultivar “Kigokoro85” (Takii & Co.) were sown in a seedling cultivation cell tray (vegetable tray [25×25‍ ‍mm, 200 cells]; Yanmar Holdings) under greenhouse conditions on September 9, 2020 and grown for 31 days. Takii cell baido TM-1 (Takii & Co.) was used as the planting soil (3 L tray^–1^). Seedlings were planted in CO and CK plots fertilized with a compound fertilizer and Chiyoda-Kasei, respectively, in an experimental field (light-colored Andosol, 36°01′07″N, 139°41′27″E, 9.4 m a.s.l.) (Shiraoka). Inter-row and intra-row distances were 60 and 40‍ ‍cm, respectively. Row lengths were both 10 m for the CO and CK plots (24 m^2^ in size), and planting was performed on October 6, 2020.

A basal fertilization (140‍ ‍kg of N ha^–1^, 140‍ ‍kg of P_2_O_5_ ha^–1^, and 140‍ ‍kg of K_2_O kg ha^–1^, a compound fertilizer) was applied to the CO and CK plots at the time of planting. Seedlings and rows were covered with a mulching film (L-L strengthened Sankyo mulch, dark green, thickness of 0.02‍ ‍mm, width of 95‍ ‍cm; Sankyo). A compound fertilizer (60‍ ‍kg of N ha^–1^ [93% ammonia nitrogen {w/w} and 7% urea {w/w}], 60‍ ‍kg of P_2_O_5_ ha^–1^, and 60‍ ‍kg of K_2_O kg ha^–1^) and Chiyoda-kasei (60‍ ‍kg of N ha^–1^ [100% ammonia nitrogen {w/w}], 60‍ ‍kg of P_2_O_5_ ha^–1^, and 40‍ ‍kg of K_2_O kg ha^–1^) were used as additional fertilizations for the CO and CK plots, respectively. Additional fertilizations were applied twice to the soil surface of each plot, 20 to 30‍ ‍cm from a plant under a mulch film, on October 26 and November 17, 2020.

The short diameters of 22 and 24 heads of napa cabbage were measured for the CO and CK plots, respectively, on November 5, 2020. Growth parameters (the weight of all above-ground tissue, the head, and outer leaves, and the ratio to head weight) were examined for 10 plants randomly selected from each of the CO and CK plots on December 18, 2020. Head weight was calculated as follows: (weight of all above-ground tissue)–(weight of the outer leaves). The ratio to head weight was calculated as follows: (head weight) (weight of all above-ground tissue)^–1^×100. Based on the head weight, four representative heads from 10 plants described above were selected from each of the CO and CK plots and the corresponding roots of 4 plants per plot were carefully dug up as replicates for DNA extraction on December 18, 2020. After serially washing and rinsing roots with tap and sterilized water, roots were stored at –30°C until used for DNA extraction.

The roots of individual plants were grounded in liquid nitrogen with a mortar and pestle. A portion of a pulverized sample (0.4 g) was transferred to a Lysing Matrix E tube (MP Biomedicals), and a DNA sample was prepared as described in a previous study (Ikeda *et al.*, 2004), except that a homogenizer (FastPrep^®^24, MP Biomedicals) was used for the bead-beating step (5.5 ms^–1^ at room temperature for 30 s) and a DEAE-cellulose column treatment was omitted. Pelleted DNA was then washed with 70% ethanol and suspended in 100‍ ‍μL of TE buffer (pH 7.6).

To conduct a community ana­lysis of napa cabbage root-associated bacteria and fungi, PCR amplification of the V3–V4 region of the bacterial 16S rRNA gene and the partial sequence of the internal transcribed space in the fungal rRNA gene region, sequencing with a MiSeq sequencer (Illumina), and sequence editing with Qiime (Caporaso *et al.*, 2010) and Qiime2 (Bolyen *et al.*, 2019) were conducted at the Bioengineering Lab prior to statistical ana­lyses, as summarized in the supplementary materials. Statistical ana­lyses were performed using JMP software version 12 (SAS Institute). Raw reads used in the present study were deposited into the NCBI SRA database (BioProject accession number: PRJNA718730).

The short diameter of the head, the weight of all above-ground tissue, head weight, and the ratio to head weight were significantly higher in the CK plot than in the CO plot (Table 1). The results of soil chemical ana­lyses showed that ammonia nitrogen was 3-fold higher in the CK plot than in the CO plot, suggesting the high diffusibility of nitrogen from the surface of the field into soil in the CK plot (Table S1). No significant differences were observed in any diversity indexes between the CO and CK plots (Table S2).

Taxonomic ana­lyses of bacterial sequence data identified 20 phyla, 50 classes, 82 orders, 129 families, 200 genera, and 217 species. A clustering ana­lysis of bacterial sequence data with 100% identity generated 574 ASVs. Among these taxa and ASVs, 10 taxa and 5 ASVs were identified as bacterial groups that were significantly less abundant in the CK plot than in the CO plot (Table 2). Most of the closest known bacterial groups of these taxa and ASVs were previously reported to be sensitive to nitrogen fertilization and/or related to the geochemical cycles of nitrogen. *Caulobacteraceae* and *Thermomonosporaceae* were previously shown to reduce their abundance in response to an increase in nitrogen fertilization in the rhizosphere soil of canola (*B. napus*) (Monreal *et al.*, 2018). *Caulobacteraceae* and *Chitinophagaceae* increased their abundance in the rhizosphere soil and roots of *Triticum aestivum* under nitrogen starvation conditions (Pagé *et al.*, 2019). The direct application of nitrate to soil decreased the abundance of *Caulobacteraceae* in *Arabidopsis* roots (Konishi *et al.*, 2017). Collectively, these findings suggest that *Caulobacteraceae* favors a low soil nitrogen level. *Caulobacter* and *Chitinophagaceae* have both been recognized as beneficial bacterial groups (Madhaiyan *et al.*, 2015; Luo *et al.*, 2019), and may not be competitive in a rhizosphere under a high soil nitrogen level.

*Kribbella* and *Niastella* (corresponding to ASV-B3 in Table S3) have been identified as a nitrate reducer and denitrifier, respectively (Nishizawa *et al.*, 2014; Ozdemir-Kocak *et al.*, 2017). Increases in the abundance and activities of these bacteria in a rhizosphere are not favorable from the viewpoint of agriculture because their effects on mineral nitrogen may reduce the efficiency of nitrogen fertilization. *Niastella* has also been reported as a bacterial group associated with the roots of sugarcane with relatively high abundance, and a root-associated OTU belonging to this genus was enriched under lower nitrogen fertilization than the standard level (Yeoh *et al.*, 2016). *Rubrivivax* is a nitrogen fixer (Willems *et al.*, 1991) and is likely to be less competitive in nitrogen-rich environments. *Variovorax* (corresponding to ASV-B5 in Table S3) is a well-known beneficial bacterial group that exerts plant growth-promoting (PGP) effects on diverse plants, including *Brassica* species (Bertrand *et al.*, 2001; Natsagdorj *et al.*, 2019; Okazaki *et al.*, 2021). An ASV belonging to *Variovorax* was previously shown to be enriched in the roots of wheat under low nitrogen fertilization (Pagé *et al.*, 2019). Furthermore, *Dokdonella* (corresponding to ASV-B11) decreased its abundance under high nitrogen fertilization (Shang and Yi, 2015).

As bacterial groups that were more abundant in the CK plot than in the CO plot, 2 taxa and 6 ASVs were identified (Table 2). Although root-*Legionellales* interactions remain largely unknown, regarding nitrogen fertilization, Zhou *et al.* (2015) reported that the relative abundance of *Legionellales* positively correlated with nitrogen fertilization and wheat yield. They also linked their abundance to the high yield of wheat, which was associated with the high capability for ammonium assimilation by members of *Legionellales* (Vishnivetskaya *et al.*, 2013). Consistent with these findings, the high abundance of unclassified *Legionellales* in the CK plot appeared to be attributed to responses to the high concentration of ammonia nitrogen in the CK plot (Table S1). *Chitinophaga filiformis* (corresponding to ASV-B2 in Table S3) is a chitinolytic bacterium (Kämpfer *et al.*, 2006) that may be antagonistic to fungal groups such as *Bipolaris*, which has chitin as the main component of the cell wall. The responses of *Sphingobacteriales* to fertilization have been examined in studies using community ana­lyses. Sapp *et al.* (2015) showed that the relative abundance of some OTUs belonging to *Sphingobacteriales* positively or negatively correlated with N and P fertilizers at the OTU level. In a study by Ling *et al.* (2017), the relative abundance of *Sphingobacteriales* negatively and positively correlated with nitrogen and phosphate fertilization, respectively, at the order and genus levels. These findings suggest that each bacterial group of *Sphingobacteriales* may respond differently to fertilization input at lower taxonomic levels. In the present study, the taxonomic affiliation of ASV-B4 (*Sphingobacteriales*) at a lower taxonomic level was unclear, as shown in Table S3, and, thus, difficulties are associated with comparisons with previous studies and obtaining insights for future research.

*Pelomonas* (corresponding to ASV-B6 in Table S3) is a root-associated diazotroph (Xie and Yokota, 2005) that has also been identified as an endophytic bacterial group for rapeseed (Glaeser *et al.*, 2020). *Methylophilaceae* (corresponding to ASV-B8 in Table S3) has been reported to link methanol oxidation to denitrification in freshwater lake sediment. Previous studies demonstrated the emission of methanol from roots as a byproduct of active growth (Sy *et al.*, 2005) and the colonization of methanol-utilizing bacteria, such as *Methylobacterium* species, on roots (Poonguzhali *et al.*, 2008). The presence of ASV-B8 only in the CK plot may reflect the dominance of a unique bacterial group that assimilates methanol derived from actively growing roots in the CK plot in a nitrate-dependent manner.

Taxonomic ana­lyses of fungal sequence data identified 6 phyla, 9 classes, 26 orders, 49 families, and 64 genera. These results revealed that the sum of the relative abundance of *Bipolaris* and *Olpidium* was more than 90% for all samples examined. A clustering ana­lysis of fungal sequence data with 97% identity generated 419 OTUs. A total of 318 OTUs (75.9%) were counted for *Bipolaris* sp. and their abundance ranged between 0 and 50% at the individual plant level. As fungal groups that were significantly less abundant in the CK plot than in the CO plot, 5 taxa and 18 OTUs (all belonging to *Bipolaris*) were identified (Table 3). A phylogenetic ana­lysis revealed that 18 OTUs in Table 3 were largely classified into 3 groups (Fig. S2). In addition, a strong negative correlation was observed between the relative abundance of *Bipolaris*- and *Olpidium*-related taxa from the phylum to OTU levels (Fig. 1). Correlation ana­lyses at the OTU level further revealed that the relative abundance of one dominant (OTU-F13) and 34‍ ‍minor OTUs in the genus *Bipolaris* negatively correlated with that of OTU-F20 (*Olpidium*) (Table S4).

The high dominance of *Bipolaris* sp. in the roots of napa cabbage cultivated in Japan may be attributed to Andosol. While Andosol is the most common soil type for upland crops in Japan, it has rarely been found in the countries in which previous studies conducted fungal community ana­lyses of the rhizospheres of *Brassica* species (Neupane *et al.*, 2013; Lay *et al.*, 2018; Picot *et al.*, 2021). *Bipolaris* was shown to be relatively insensitive to volatile isothiocyanates (ITCs) originating from glucosinolates in the roots of *Brassica* species (Kirkegaard *et al.*, 1996). Since *Bipolaris* is one of the most important fungal pathogens for plants (Bhunjun *et al.*, 2020), further studies are warranted on this fungal group in relation to the soil type and fertilization practices. Furthermore, research involving culture-dependent methodologies is needed to reveal the ecological functionality of unknown *Bipolaris*.

As fungal groups that were significantly more abundant in the CK plot than in the CO plot, 2 taxa and 2 OTUs were identified (Table 3). *Olpidium* species are recognized as a common fungal parasite for the roots of diverse plant species, particularly in the family *Brassicaceae* (Hartwright *et al.*, 2010), and the relative abundance of *Olpidium* (69.8%) in the roots of canola was previously reported to be high (Lay *et al.*, 2018). Floc’h *et al.* (2020) showed that the low and high relative abundance of *Olpidium *positively and negatively correlated with the yields of canola and suggested the potential of a population dependent-change in the ecological role of *Olpidium* sp. Lebreton *et al.* (2019) hypothesized that for a non-mycorrhizal plant, an endophytic fungus, such as *Opidium* species, may play a role in plant nutrition uptake, similar to other crucifer plant-fungus interactions.

The relative abundance of *Olpidium* in the rhizospheres of oilseed rape and wheat was shown to increase under high nitrogen fertilization (Illescas *et al.*, 2020; Picot *et al.*, 2021). Since *Olpidium* is an obligate biotroph, it may be more susceptible to changes in the environmental conditions surrounding host plants than *Bipolaris*, which is generally considered to be a facultative fungal group. Lebreton *et al.* (2019) demonstrated that the relative abundance of an unknown *Chytridiomycota* markedly increased with a decrease in that of *Ascomycota* in the roots of napa cabbage in the later stage of vegetative growth. Therefore, the marked difference observed in relative abundance between *Bipolaris* (belonging to *Ascomycota*) and *Olpidium* (belonging to *Chytridiomycota*) in the present study may also reflect variations in the succession stages of root-associated fungal communities between the CO and CK plots towards the mature growth stage of the napa cabbage head.

Correlation ana­lyses were conducted between the growth parameters of napa cabbage and root-associated microbes listed in Tables 2 and 3 (Table 4 and Fig. S3). The results obtained revealed that ASV-B2 (*Chitinophaga*), ASV-B6 (*Comamonadaceae*), and *Xylariaceae* positively correlated with the growth parameters of napa cabbage. The closest relatives for ASV-B2 and ASV-B6 revealed by blast ana­lyses suggest that these bacterial ASVs exerted PGP effects. As microbial groups that negatively correlated with the growth parameters of napa cabbage, 3 taxa (*Alphaproteobacteria*, *Caulobacteraceae*, and *Caulobacter*) and 7 ASV or OTUs (ASV-B3 [*Niastella*] and 6 OTUs belonging to *Bipolaris*) were identified. It was reasonable that ASV-B3 (*Niastella*) negatively correlated with plant growth parameters because *Niastella* is considered to function as a denitrifier that may reduce the efficiency of fertilization.

In conclusion, the present study suggests that qualitative differences among additional fertilizers most likely cause a shift in bacterial and fungal community structures in the napa cabbage root. The results obtained herein will facilitate our understanding of the effects of fertilization practices on plant-associated microbes and provide insights into the regulation of the plant-associated microbiome in an agronomic environment.

## Citation

Ikeda, S., Okazaki, K., Tsurumaru, H., Suzuki, T., and Hirafuji, M. (2022) Effects of Different Types of Additional Fertilizers on Root-associated Microbes of Napa Cabbage Grown in an Andosol Field in Japan. *Microbes Environ ***37**: ME22013.

https://doi.org/10.1264/jsme2.ME22013

## Supplementary Material

Supplementary Material

## Figures and Tables

**Fig. 1.**
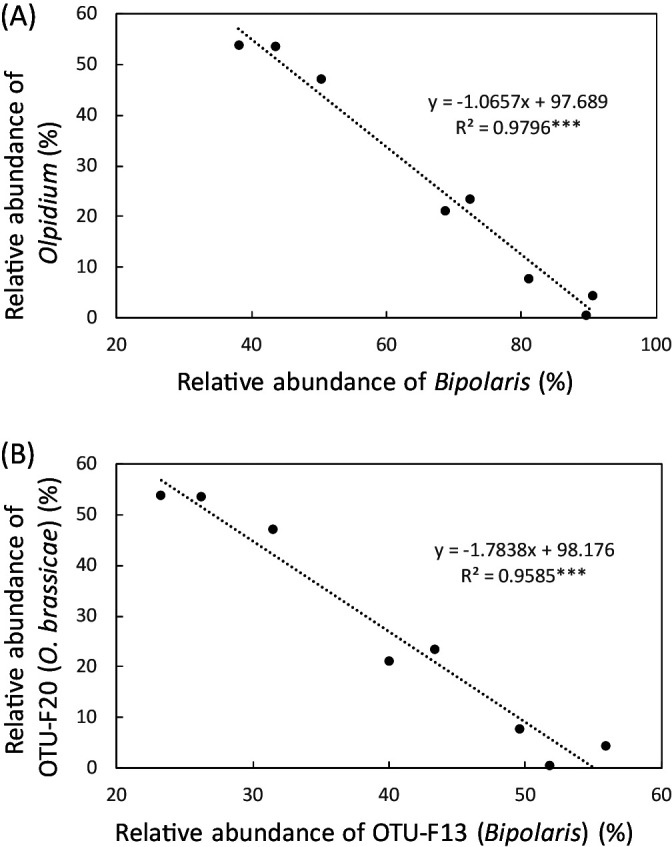
Correlation plot of the relative abundance of *Bipolaris* and *Olpidium*. Panel A: Correlation plot at the genus level. Panel B: Correlation plot at the OTU level. *** indicates a significant difference at *P*<0.001.

**Table 1.** Comparisons of growth parameters of napa cabbage heads between compound fertilizer and Chida-kasei plots

Fertilization plots^a^	Short diameter of the head (cm)	Weight of all above-ground tissue (kg plant^–1^, *n*=10)	Head weight (kg plant^–1^, *n*=10)^b^	Weight of outer leaves (kg plant^–1^, *n*=10)	Ratio to head weight in above-ground tissue (%, *n*=10)^c^
CO	52 (*n*=22)	2.52	1.57	0.95	62.2
CK	55** (*n*=24)	2.83*	1.97***	0.86	69.5**

^a^ CO and CK indicate the compound fertilizer and Chiyoda-kasei plots, respectively. ^b^ (Weight of all above-ground tissue)–(Weight of outer leaves). ^c^ (Head weight) (Weight of all above-ground tissue)^–1^×100. *, **, and *** indicate a significant difference with a *t*-test at *P*<0.05, *P*<0.01, and *P*<0.001, respectively. All traits were examined on December 18, 2020, except for the short diameter of the head, which was measured on November 5, 2020.

**Table 2.** Bacterial groups showing significant differences in relative abundance between compound fertilizer and Chiyoda-kasei plots

Taxon^a^	Relative abundance (%)^b^		Fold change (CK/CO)
	CO^c^	CK	
Class			
*Alphaproteobacteria*	14.66±0.68	12.66±1.18*	0.86
***Planctomycetia***	0±0	0.09±0.09*	—
Order			
*Rhizobiales*	7.72±0.32	6.76±0.64*	0.88
*Spirobacillales*	0.05±0.03	0±0*	—
*Sva0725*	0.27±0.22	0.04±0.09*	0.16
Family			
*Caulobacteraceae*	4.11±0.36	3.37±0.41*	0.82
***Unclassified Legionellales***	0.09±0.09	0.33±0.10*	3.55
*Thermomonosporaceae*	0.19±0.16	0±0*	—
Genus			
*Caulobacter*	3.26±0.18	2.56±0.26**	0.79
*Kribbella*	0.22±0.23	0±0*	—
*Rubrivivax*	1.61±0.82	0.37±0.67*	0.23
Species			
*Unclassified Caulobacter*	1.53±0.37	0.92±0.16*	0.60
ASV^d^			
ASV-B1 (*Chitinophagaceae*)	0.24±0.11	0.06±0.07*	0.25
**ASV-B2 (*****Chitinophaga*****)**	0.16±0.24	0.91±0.26*	5.58
ASV-B3 (*Niastella*)	0.84±0.61	0±0*	—
**ASV-B4 (*****Sphingobacteriales*****)**	0±0	0.51±0.05***	—
ASV-B5 (*Comamonadaceae*)	1.00±0.90	0±0*	—
**ASV-B6 (*****Comamonadaceae*****)**	0.18±0.36	0.68±0.13*	3.81
**ASV-B7 (*****Ellin6067*****)**	0±0	0.16±0.12*	—
**ASV-B8 (*****Methylotenera mobilis*****)**	0±0	0.20±0.16*	—
ASV-B9 (*Myxococcales*)	0.28±0.21	0±0*	—
**ASV-B10 (*****Legionellales*****)**	0.07±0.10	0.33±0.10*	4.88
ASV-B11 (*Dokdonella*)	1.08±0.11	0.77±0.14*	0.71

^a^ When the same value for relative abundance was obtained at different taxonomic levels for a bacterial group, only the lowest taxonomic group was shown. Bacterial groups showing a higher abundance in the CK plot than in the CO plot are shown in bold font. ^b^ Relative abundance (%) is calculated based on 2,927 reads per sample and the results of the average and S.D. (*n*=4) for each of the fertilization conditions are shown. ^c^ CO and CK indicate the compound fertilizer and Chiyoda-kasei plots, respectively. ^d^ The closest taxon to a representative sequence of an ASV is shown in parentheses. *, *, and *** indicate a significant difference between the CO and CK plots at *P*<0.05, *P*<0.01, and *P*<0.001, respectively.

**Table 3.** Fungal groups showing significant differences in relative abundance between compound fertilizer and Chiyoda-kasei plots

Taxon^a^	Relative abundance (%)^b^		Fold change (CK/CO)
	CO^c^	CK	
Family			
*Pleosporaceae*	83.9±8.6	50.5±13.0**	0.60
***Xylariaceae***	0±0	0.12±0.09*	—
Genus			
*Bipolaris*	83.5±8.6	50.2±13.3**	0.60
***Olpidium***	9.1±10.0	43.9±15.4**	4.84
OTU^d^			
OTU-F1 (*Bipolaris*)	1.60±0.29	0.97±0.35*	0.61
OTU-F2 (*Bipolaris*)	0.47±0.12	0.26±0.10*	0.55
OTU-F3 (*Bipolaris*)	0.63±0.10	0.26±0.05***	0.40
OTU-F4 (*Bipolaris*)	0.82±0.24	0.43±0.18*	0.53
OTU-F5 (*Bipolaris*)	0.19±0.07	0.08±0.02*	0.43
OTU-F6 (*Bipolaris*)	0.04±0	0.01±0.02*	0.25
OTU-F7 (*Bipolaris*)	2.74±0.05	1.82±0.35**	0.66
OTU-F8 (*Bipolaris*)	0.04±0.03	0±0*	—
OTU-F9 (*Bipolaris*)	0.64±0.17	0.34±0.13*	0.52
OTU-F10 (*Bipolaris*)	0.13±0.07	0.03±0.03*	0.20
OTU-F11 (*Bipolaris*)	0.07±0.03	0.01±0.02**	0.13
OTU-F12 (*Bipolaris*)	0.29±0.08	0.11±0.06*	0.36
OTU-F13 (*Bipolaris*)	50.14±5.24	30.25±7.36**	0.60
OTU-F14 (*Bipolaris*)	0.18±0.06	0.07±0.04*	0.40
OTU-F15 (*Bipolaris*)	0.09±0.06	0.02±0.02*	0.20
OTU-F16 (*Bipolaris*)	0.06±0.02	0.04±0*	0.57
OTU-F17 (*Bipolaris*)	0.41±0.05	0.19±0.03***	0.46
OTU-F18 (*Bipolaris*)	0.04±0.03	0±0*	—
**OTU-F19 (*****Podospora*****)**	0±0	0.30±0.24*	—
**OTU-F20 (*****Olpidium brassicae*****)**	9.07±10.0	43.9±15.4**	4.84

^a^ When the same or a similar value for relative abundance was obtained at different taxonomic levels for a fungal group, only the lowest taxonomic group was shown. Fungal groups showing a higher abundance in the CK plot than in the CO plot are shown in bold font. ^b^ Relative abundance (%) is calculated based on 2,835 reads per sample and the results of the average and S.D. (*n*=4) for each of the fertilization conditions are shown. ^c^ CO and CK indicate the compound fertilizer and Chiyoda-kasei plots, respectively. ^d^ The closest taxon to the sequence of an OTU is shown in parentheses. *, *, and *** indicate a significant difference between the CO and CK plots at *P*<0.05, *P*<0.01, and *P*<0.001, respectively.

**Table 4.** Relationships between plant growth parameters of napa cabbage and the relative abundance of napa cabbage root-associated microbes showing a significant difference between compound fertilizer and Chiyoda-kasei plots

Taxon^a^	Weight of all above-ground tissue^b^			Head weight			Ratio to head weight in above-ground tissue	
	P, N	R^2^		P, N	R^2^		P, N	R^2^
Bacteria								
*Alphaproteobacteria*	N	0.42		N	0.56*		N	0.41
*Caulobacteraceae*	N	0.43		N	0.57*		N	0.46
*Caulobacter*	N	0.54*		N	0.77**		N	0.68*
**ASV-B2 (*****Chitinophaga*****)**	P	0.17		P	0.47		P	0.78**
ASV-B3 (*Niastella*)	N	0.52*		N	0.59*		N	0.45
**ASV-B6 (*****Comamonadaceae*****)**	P	0.53*		P	0.53*		P	0.31
Fungi								
***Xylariaceae***	P	0.43		P	0.56*		P	0.36
OTU-F2 (*Bipolaris*)	N	0.69*		N	0.64*		N	0.27
OTU-F3 (*Bipolaris*)	N	0.23		N	0.50*		N	0.70**
OTU-F4 (*Bipolaris*)	N	0.19		N	0.42		N	0.61*
OTU-F5 (*Bipolaris*)	N	0.46		N	0.54*		N	0.44
OTU-F8 (*Bipolaris*)	N	0.001		N	0.15		N	0.58*
OTU-F15 (*Bipolaris*)	N	0.50		N	0.59*		N	0.50

^a^ Microbial groups showing a significantly higher abundance in the CK plot (Chiyoda-kasei) than in the CO plot (a compound fertilizer) are shown in bold font. ^b^ P, N, and R^2^ indicate a positive correlation, negative correlation, and decision coefficient, respectively. * and ** indicate a significant difference at *P*<0.05 and *P*<0.01, respectively. Correlations based on a confidence curve of the regression line at *P*<0.05 are highlighted in gray.
