# Supplementary material for: Effects of Different Types of Additional Fertilizers on Root-associated Microbes of Napa Cabbage Grown in an Andosol Field in Japan
Source: Microbes Environ. 2022 May 31;37(2):ME22013. doi: 10.1264/jsme2.ME22013 (PMC9530736; doi:10.1264/jsme2.ME22013)
Supplement: Supplementary file 1 — Supplementary Material [file 37_22013_s1.pdf]

Supplementary materials

Title: Effects of different types of additional fertilizers on root-associated microbes of napa cabbage grown in an Andosol field in Japan

Authors: Seishi Ikeda[1]*, Kazuyuki Okazaki[1], Hirohito Tsurumaru[2], Takanori Suzuki[3], and Masayuki Hirafuji[4]

Author affiliation: [1]Memuro Research Station, Hokkaido Agricultural Research Center, National Agriculture and Food Research Organization, 9-4 Shinsei-minami, Memuro, Kasai-gun, Hokkaido 082-0081, Japan; [2]Faculty of Agriculture, Kagoshima University, 1-21-24, Korimoto, Kagoshima 890-0065, Japan; [3]SUN AGRO Co., Ltd., 1470 Shiraoka, Shiraoka, Saitama 349-0218, Japan; [4]The University of Tokyo, 1-1-1, Midoricho, Nishi-tokyo, Tokyo, 183-0002, Japan

*__Corresponding author__: Seishi Ikeda, Memuro Research Station, Hokkaido Agricultural Research Center, National Agriculture and Food Research Organization, 9-4 Shinsei-minami, Memuro, Kasai-gun, Hokkaido 082-0081, Japan. E-mail: sikeda67@affrc.go.jp; Tel.: +81-155-62-9276; Fax: +81-155-61-2127.

Materials and Methods

Soil chemical analyses

Soil samples (between 5 cm and 15 cm depth) prior to planting seedlings were collected from 5 places (center and 4 corners on the diagonal line drawn in the experimental field) by using an auger and were combined as a composite soil sample on June 29, 2020 after potato cultivation. Soil samples after sampling of heads and roots of napa cabbage were collected from 3 places of intra-row spaces and were combined as a composite soil sample for each of CO and CK plots on December 23, 2020. The analyses of chemical characteristics for field soils used in the present study were carried out by employing the following methods: pH (H_2O) and pH (KCl); pH meter, EC; EC meter, NH_4-N; indophenol method, NO_3-N; hydrazine reduction method, P_2O_5; Murphy-Riley method, K_2O; Flame photometry, MgO; Xylidil blue method, CaO; o-cresolphthalein complexon method, Phosphate absorption coefficient; Ammonium vanadomolybdate absorption photometry (30 min buffer extraction instead of 24 hours buffer extraction), and CEC; Indophenol method (simplified method). All analyses,

except pH and EC, were carried out by using a Soil & Plant Analyzer Development SFP-4i (Fujihira Industry Co., Ltd, Tokyo, Japan).

Sequencing and editing of sequence data prior to statistical analyses

Briefly, PCR amplification of a partial sequence of bacterial 16S rRNA gene was conducted using the 1st-515f_MIX and 1st-806r_MIX primer set that amplified the V3-V4 region of the bacterial 16S rRNA gene (Caporaso *et al*. 2011). PCR amplification for bacterial 16S rRNA gene was performed as follows: 94°C for 2 min initially, 30 cycles of a program (94°C for 30 s, 50°C for 30 s, and 72°C for 30 s) and then final extension at 72°C for 5 min. PCR amplification for fungal ITS was conducted using the 1st-gITS and 1st-gITS4 primer set that amplified the internal transcribed sequence of the fungal 18S rRNA gene region (Ihrmark *et al*. 2012). PCR amplification for fungal ITS was performed as follows: 94°C for 2 min initially, 30 cycles of a program (94°C for 30 s, 50°C for 30 s, and 72°C for 60 s) and then final extension at 72°C for 5 min. PCR amplicons of bacterial 16S rRNA gene and fungal ITS were used as a template DNA for the second PCR and paired-end sequence (2 ×300 bp) on a MiSeq sequencer using a MiSeq Reagent Kit v3 (Illumina K.K., Tokyo, Japan) as described by Nakamura *et al*. (2020).

For bacterial 16S rRNA gene, extraction of raw reads containing a perfect primer sequence, removal of primer and 50 bases of 3’end sequences, and trimming (minimum length 40 bases; minimum average quality score 20) were carried out as described by Nakamura *et al*. (2020). Trimmed reads were merged (minimum overlap 10 bases; average merged length 250 bases; average length of individual reads 230 bases) using the FLASH software (Magoc and Salzberg, 2011). Taxonomic assignment for the merged sequences was carried out using a script of Qiime (Caporaso *et al*., 2010), and sequences classified into archaea, chloroplast, mitochondria, and unassigned sequences were removed. Then, removal of chimera and noise sequences and generation of ASVs and their representative sequences were carried out with data2 Qiime2 plugin (Bolyen *et al*., 2019). Taxonomic assignment for the representative sequences was carried out with feature-classifier Qiime2 plugin by comparing with 97% OTUs of Greengene. Alpha-diversity measures were carried out with a script of Qiime.

For fungal ITS, extraction of raw reads containing a perfect primer sequence, removal of primer sequences, and trimming (minimum length 40 bases; minimum average quality score 20) were carried out as described by Nakamura *et al*. (2020). Trimmed reads were merged (minimum overlap 10 bases; average merged length 320 bases; average length of individual reads 280 bases) using the FLASH software. For

unmerged reads, 50 bases of 3’end were deleted and merged again as described above. These processes for unmerged reads were repeated another twice. A chimera check of these sequences was performed via the UCHIME algorithm in the USEARCH package (Edgar, 2010; Edgar *et al.*, 2011). A database for chimera check was 97% OTU of UNITE. Taxonomic assignment for the non-chimera sequences was carried out using a script of Qiime, and sequences classified into unassigned sequences were removed. Then, generation of OTUs with 97% identity and taxonomic assignment for the representative sequences of OTUs were carried out with a workflow script of Qiime (no reference, default parameters). Alpha- and beta-diversity measures were carried out with a script of Qiime.

Phylogenetic tree analyses

Sequences were aligned using the CLUSTAL W program. The neighbor-joining method was used to build the trees (Saitou and Nei, 1987). The PHYLIP format tree output was applied using the bootstrapping procedure with 1,000 replicates (Felsenstein, 1985). The tree was constructed with TreeView software (Page, 1996).

References

Bolyen, E., Rideout, J.R., Dillon, M.R., Bokulich, N.A., Abnet, C.C., Al-Ghalith, G.A., *et al*. (2019) Reproducible, interactive, scalable and extensible microbiome data science using QIIME 2. *Nat Biotechnol* **37**: 852–857.

Caporaso, J.G., Kuczynski, J., Stombaugh, J., Bittinger, K., Bushman, F.D., Costello, E.K., *et al*. (2010) QIIME allows analysis of high-throughput community sequencing data. *Nat Methods* **7**: 335–336.

Caporaso J.G., Lauber, C.L., Walters, W.A., Berg-Lyons, D., Lozupone, C.A., Turnbaugh, P.J., *et al*. (2011) Global patterns of 16S rRNA diversity at a depth of millions of sequences per sample. *Proc Natl Acad Sci USA* **108 Suppl 1**: 4516–4522.

Edgar, R.C. (2010) Search and clustering orders of magnitude faster than BLAST. *Bioinformatics* **26**: 2460–2461.

Edgar, R.C., Haas, B.J., Clemente, J.C., Quince, C., and Knight, R. (2011) UCHIME improves sensitivity and speed of chimera detection. *Bioinformatics* **27**: 2194–2200.

Felsenstein, J. (1985) Confidence limits on phylogenies: an approach using the bootstrap. *Evolution* **39**: 783–791.

Ihrmark, K., Bödeker, I.T.M., Cruz-Martinez, K., Friberg, H., Kubartova, A., Schenck, J., *et al*. (2012) New primers to amplify the fungal ITS2 region – evaluation by 454-sequencing of artificial

and natural communities. *FEMS Microbiol Ecol* **82**: 666–677.

Magoc, T., and Salzberg, S.L. (2011) FLASH: fast length adjustment of short reads to improve genome assemblies. *Bioinformatics* **27**: 2957–2963.

Nakamura, Y., Ishibashi, M., Kamitani, Y. and Tsurumaru, H. (2020) Microbial community analysis of digested liquids exhibiting different methane production potential in methane fermentation of swine feces. *Appl Biochem Biotechnol* **191**: 1140–1154.

Page, R.D. (1996) Tree View: An application to display phylogenetic trees on personal computers. *Bioinformatics* **12**: 357–358.

Saitou, N., and Nei, M. (1987) The neighbor-joining method: a new method for reconstructing phylogenetic trees. *Mol Biol Evol* **4**: 406–425.

Table S1. Chemical characteristics of field soils used in the present study

Analysis items[a]	Fertilization plots[b]		
	Prior to planting (2020/6/26)[c]	CO (2020/12/23)	CK (2020/12/23)
pH (H_2O)	6.21	5.38	5.64
pH (KCl)	5.25	4.92	4.99
EC (1:5)	0.07	0.94	1.292
NH_4-N	23.3	213	686
NO_3-N	3.5	210	192
Available P_2O_5	418	360	340
Exchangeable K_2O	316	525	716
Exchangeable MgO	465	567	596
Exchangeable CaO	3128	3290	3700
Base saturation (%)	77.6	86	97.1
Phosphate absorption coefficient	1900	-	-
CEC (me kg^{-1})	182	-	-

[a]Data for NH_4-N, NO_3-N, available P_2O_5, exchangeable K_2O, exchangeable MgO, and exchangeable CaO are expressed as mg kg^{-1}.

[b]CO and CK stand for a compound fertilizer and Chiyoda-kasei, respectively.

[c]Sampling dates.

Table S2. Diversity indexes for root-associated bacteria and fungi of napa cabbage grown with two different types of additional fertilizations

Microbes/ Fertilization[a]	No. of singletons[b]	No. of ASVs or OTUs[c]	Chao1	ACE	Shannon	Simpson
Bacteria						
CO	1.8 ± 3.5	152.75 ± 8.3	153.5 ± 9.7	153.3 ± 9.3	6.7 ± 0.05	0.987 ± 0.0005
CK	3.5 ± 2.5	161.25 ± 8.8	162.1 ± 9.6	162.2 ± 9.5	6.8 ± 0.10	0.988 ± 0.0011
Fungi						
CO	65.3 ± 4.2	148.5 ± 18.7	246.0 ± 38.6	247.7 ± 18.5	3.3 ± 0.5	0.70 ± 0.05
CK	69.8 ± 13.9	149.8 ± 28.5	264.4 ± 73.3	264.7 ± 68.2	3.2 ± 0.6	0.71 ± 0.06

[a]CO and CK stand for a compound fertilizer and Chiyoda-kasei, respectively.

[b]Diversity indexes are caluculated based on 2927 and 2835 reads per sample for bacteria and fungi, respectively, and the results of average and S.D. (n=4) are shown.

[c]ASVs and OTUs were shown for bacteria and fungi, respectively.

Table S3. Results of Blast analyses with representative seqences of microbial ASVs or OTUs showing statistical difference for the relative abundance between a compound fertilizer and Chiyoda-kasei

OTU[a]	Length (base)	Closest known species (Acession No.)	Score (bit)	Expect	Identity (%)
Bacteria					
ASV-B1(*Chitinophagaceae*)[b]	253	*Mucibacter soli* (NR_165710)	412	6E-111	96
**ASV-B2 (*Chitinophaga*)**	253	*Chitinophaga filiformis* (JX280517)	453	2E-123	100
ASV-B3 (*Niastella*)	253	*Niastella gongjuensis* (NR_137250)	453	7E-123	99
**ASV-B4 (*Sphingobacteriales*)**	253	*Solitalea koreensis* (NR_044568)	314	5E-81	88
ASV-B5 (*Comamonadaceae*)	253	*Variovorax boronicumulans* (MN826549)	457	4E-125	100
	253	*Variovorax paradoxus* (CP045644)	457	4E-125	100
	253	*Variovorax ginsengisoli* (MT415119)	457	4E-125	100
	253	*Variovorax rhizosphaerae* (NR_169352)	457	4E-125	100
**ASV-B6 (*Comamonadaceae*)**	253	*Pelomonas saccharophila* (NR_114189)	425	2E-115	98
**ASV-B7 (*Ellin6067*)**	253	*Sulfuritortus calidifontis* (NR_156937)	385	2E-103	94
	253	*Thauera humireducens* (NR_109534)	385	2E-103	94
**ASV-B8 (*Methylotenera mobilis*)**	253	*Methylophilus leisingeri* (NR_041258)	457	4E-125	100
	253	*Methylophilus luteus* (NR_116847)	457	4E-125	100
	253	*Methylophilus flavus* (NR_104519)	457	4E-125	100
	253	*Methylophilus rhizosphaerae* (NR_116239)	457	4E-125	100
ASV-B9 (*Myxococcales*)	253	*Kofleria flava* (NR_04198)	358	3E-95	91
	253	*Haliangium tepidum* (NR_024781)	358	3E-95	91
**ASV-B10 (*Legionellales*)**	253	*Marinobacterium zhoushanense* (NR_151997)	335	3E-88	89
	253	*Nitrincola lacisaponensis* (NR_042984)	335	3E-88	89
ASV-B11 (*Dokdonella*)	253	*Dyella monticola* (NR_171458)	403	8E-109	95
Fungi					
OTU-F1 (*Bipolaris*)	238	*Aspergillus flavus* (CP051057)	283	7E-72	79
OTU-F2 (*Bipolaris*)	238	*Aspergillus flavus* (CP051057)	306	6E-79	81
OTU-F3 (*Bipolaris*)	273	*Aspergillus flavus* (CP051057)	372	6E-99	89
OTU-F4 (*Bipolaris*)	238	*Aspergillus flavus* (CP051057)	288	2E-73	80
OTU-F5 (*Bipolaris*)	282	*Aspergillus flavus* (CP051057)	469	1E-127	97
OTU-F6 (*Bipolaris*)	283	*Aspergillus flavus* (CP051057)	476	7E-130	98
OTU-F7 (*Bipolaris*)	238	*Aspergillus flavus* (CP051057)	283	7E-72	79
OTU-F8 (*Bipolaris*)	273	*Aspergillus flavus* (CP051057)	359	1E-94	88
OTU-F9 (*Bipolaris*)	282	*Aspergillus flavus* (CP051057)	455	7E-124	96
OTU-F10 (*Bipolaris*)	282	*Aspergillus flavus* (CP051057)	469	1E-127	97
OTU-F11 (*Bipolaris*)	282	*Aspergillus flavus* (CP051057)	473	2E-129	97
OTU-F12 (*Bipolaris*)	303	*Aspergillus flavus* (CP051057)	367	3E-97	87
OTU-F13 (*Bipolaris*)	282	*Aspergillus flavus* (CP051057)	505	1E-138	99
OTU-F14 (*Bipolaris*)	282	*Aspergillus flavus* (CP051057)	482	5E-132	98
OTU-F15 (*Bipolaris*)	238	*Aspergillus flavus* (CP051057)	290	5E-74	80
OTU-F16 (*Bipolaris*)	281	*Aspergillus flavus* (CP051057)	465	1E-126	97
OTU-F17 (*Bipolaris*)	282	*Aspergillus flavus* (CP051057)	464	1E-126	96
OTU-F18 (*Bipolaris*)	282	*Aspergillus flavus* (CP051057)	473	2E-129	97
**OTU-F19 (*Podospora*)**	246	*Podospora intestinacea* (MN341351)	408	7E-110	97
**OTU-F20 (*Olpidium brassicae*)**	345	*Olpidium brassicae* (AB205212)	623	3E-174	100

[a]Micobial groups showing higher abundance in CK plot (Chiyodakasei) than in CO plot (a compound fertiizer) are shown as bold font.

[b]Closest taxon to a representative sequence of an ASV or OTU is shown in parentheses.

Table S4. *Bipolaris*-related OTUs showing significant negative correlations with OTU-F20 (*Olpidium*) and thier relative abundances in a compound fertilizer and Chiyoda-kasei plots

OTU ID	Relative abundance (%)[a]		R^{2c}
	CO	CK[b]	
OTU-F1	1.60 ± 0.29	0.97 ± 0.35*	0.70 **
OTU-F3	0.63 ± 0.10	0.26 ± 0.05***	0.82 **
OTU-F4	0.82 ± 0.24	0.43 ± 0.18*	0.74 **
OTU-F6	0.04 ± 0	0.01 ± 0.02*	0.87 ***
OTU-F7	2.74 ± 0.05	1.82 ± 0.35**	0.85 **
OTU-F8	0.04 ± 0.03	0 ± 0*	0.57 *
OTU-F9	0.64 ± 0.17	0.34 ± 0.13*	0.80 **
OTU-F10	0.13 ± 0.07	0.03 ± 0.03*	0.77 **
OTU-F11	0.07 ± 0.03	0.01 ± 0.02**	0.88 ***
OTU-F12	0.29 ± 0.08	0.11 ± 0.06*	0.73 **
OTU-F13	50.14 ± 5.24	30.25 ± 7.36**	0.96 ***
OTU-F14	0.18 ± 0.06	0.07 ± 0.04*	0.80 **
denovo8	0.48 ± 0.12	0.30 ± 0.23	0.58 *
denovo13	0.10 ± 0.04	0.04 ± 0.07	0.49 **
denovo72	0.28 ± 0.09	0.18 ± 0.04	0.58 *
denovo119	0.04 ± 0.03	0 ± 0	0.53 *
denovo128	2.72 ± 0.61	1.23 ± 0.54	0.87 ***
denovo158	0.10 ± 0.08	0.04 ± 0.04	0.47 **
denovo166	0.72 ± 0.18	0.34 ± 0.25	0.89 ***
denovo174	0.03 ± 0.02	0 ± 0	0.69 *
denovo181	0.07 ± 0.05	0.01 ± 0.02	0.58 *
denovo193	0.17 ± 0.07	0.07 ± 0	0.69 *
denovo195	0.23 ± 0.12	0.14 ± 0.10	0.62 *
denovo215	0.90 ± 0.16	0.56 ± 0.22	0.80 **
denovo260	0.21 ± 0.13	0.11 ± 0.03	0.61 *
denovo261	0.66 ± 0.16	0.38 ± 0.25	0.58 *
denovo280	0.30 ± 0.18	0.14 ± 0.08	0.52 *
denovo284	0.10 ± 0.04	0.05 ± 0.05	0.65 *
denovo327	0.22 ± 0.12	0.12 ± 0.06	0.54 *
denovo337	0.10 ± 0.03	0.04 ± 0.07	0.74 **
denovo340	6.09 ± 0.87	4.11 ± 1.66	0.86 ***
denovo359	0.56 ± 0.14	0.36 ± 0.17	0.72 **
denovo369	0.07 ± 0.06	0.04 ± 0.03	0.53 *
denovo390	0.23 ± 0.15	0.06 ± 0.05	0.62 *
denovo394	0.50 ± 0.14	0.33 ± 0.19	0.69 *

[a]Relative abundance (%) is caluculated based on 2835 reads per sample and the results of average and S.D. (n=4) for each of a compound fertilizer (CO) and Chiyoda-kasei (CK) plots are shown.

[b]*, *, and *** indicate statistical significance between CO and CK plots at $P < 0.05$, $P < 0.01$, and $P < 0.001$, respectively.

[c]R^2 stands for decision coefficient. *, *, and *** indicate statistical significance of negative correlations between *Bipolaris*-related OTUs and OTU-F20 (*Olpidium*) at $P < 0.05$, $P < 0.01$, and $P < 0.001$, respectively.

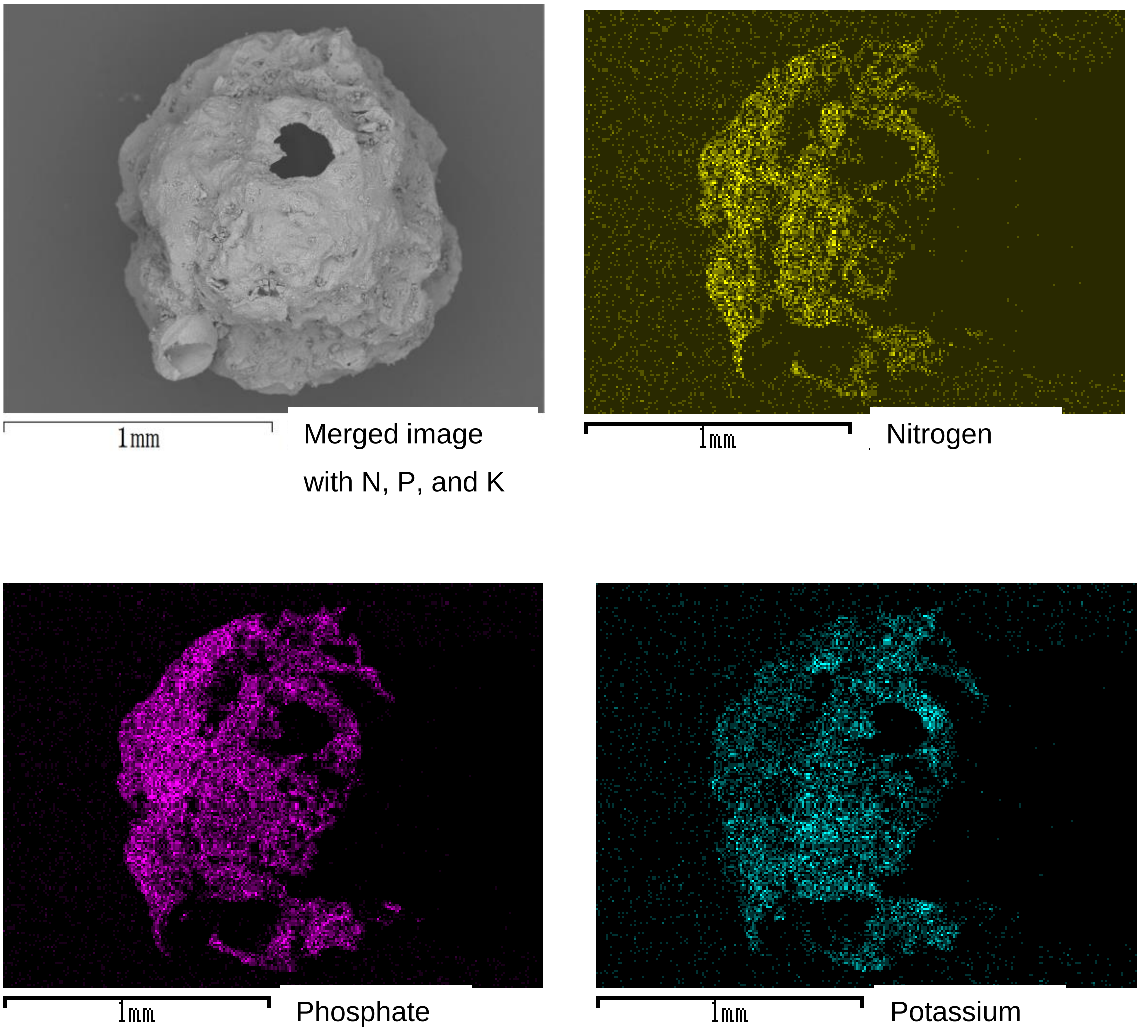

Fig. S1-A. Distribution of macronutrients on a particle of Chiyoda-kasei. Distribution of macronutrients of N, P, and K on the surface of Chiyoda-kasei particle was visualized by Energy Dispersive X-ray spectroscopy (EDX) (HITACHI, Miniscope TM3000 equipped with Swift ED 3000) (acceleration voltage: 15.0 kV and resolution: 256 x 192 pixels). Prior to the observation, the particle of Chiyoda-kasei was not sliced due to its hollow structure, but an intact particle was used for the analyses. The high similarities among nitrogen, phosphate, and potassium images suggest that each of macronutrients evenly distribute in a particle of Chiyoda-kasei.

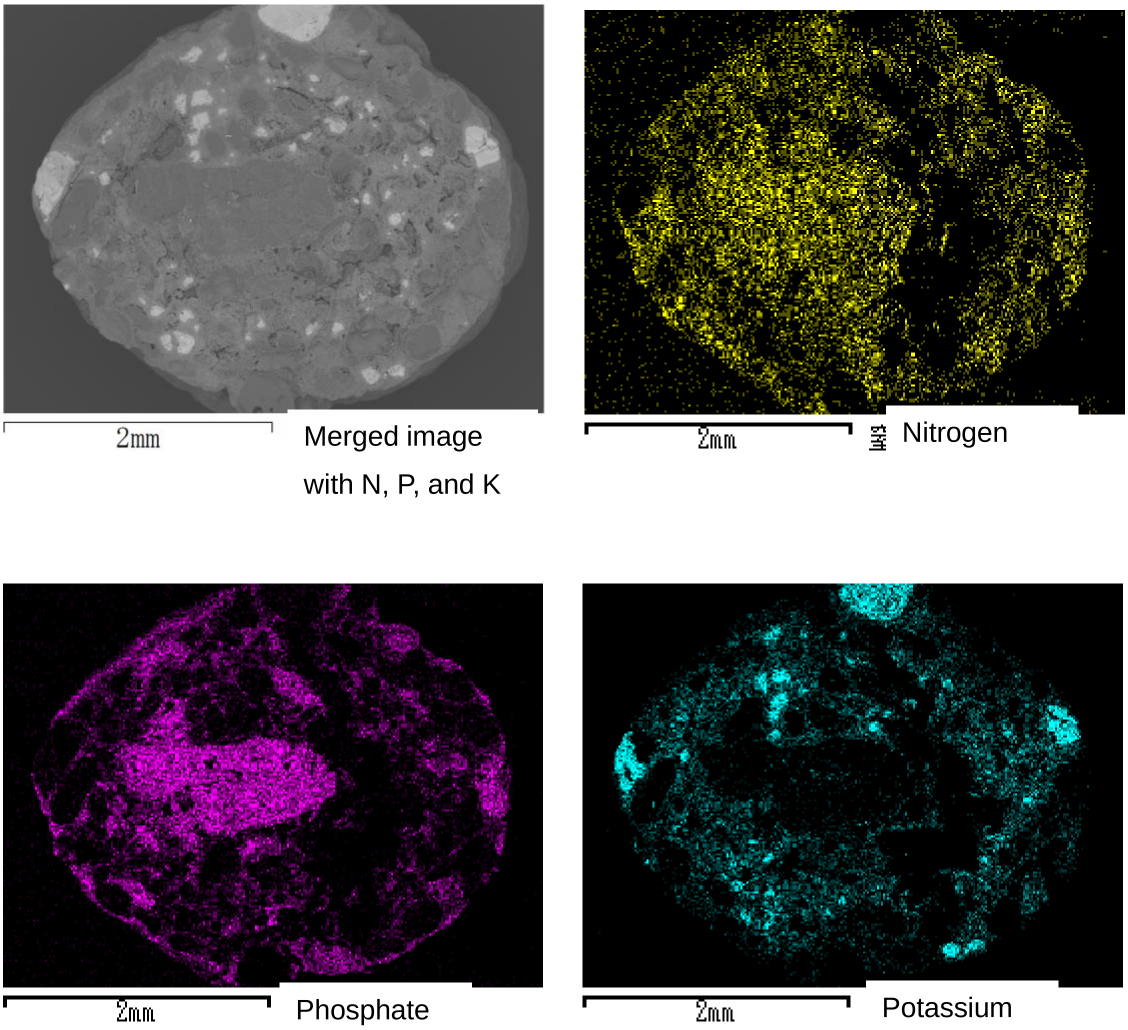

Fig. S1-B. Distribution of macronutrients in a particle of a compound fertilizer. A particle of a compound fertilizer was sliced and distribution of macronutrients of N, P, and K on the surface of slice was visualized by Energy Dispersive X-ray spectroscopy (EDX) (HITACHI, Miniscope TM3000 equipped with Swift ED 3000) (acceleration voltage: 15.0 kV and resolution: 256 x 192 pixels). The drastic dissimilarities among nitrogen, phosphate, and potassium images suggest that each of macronutrients unevenly distribute in a particle of a general compound fertilizer.

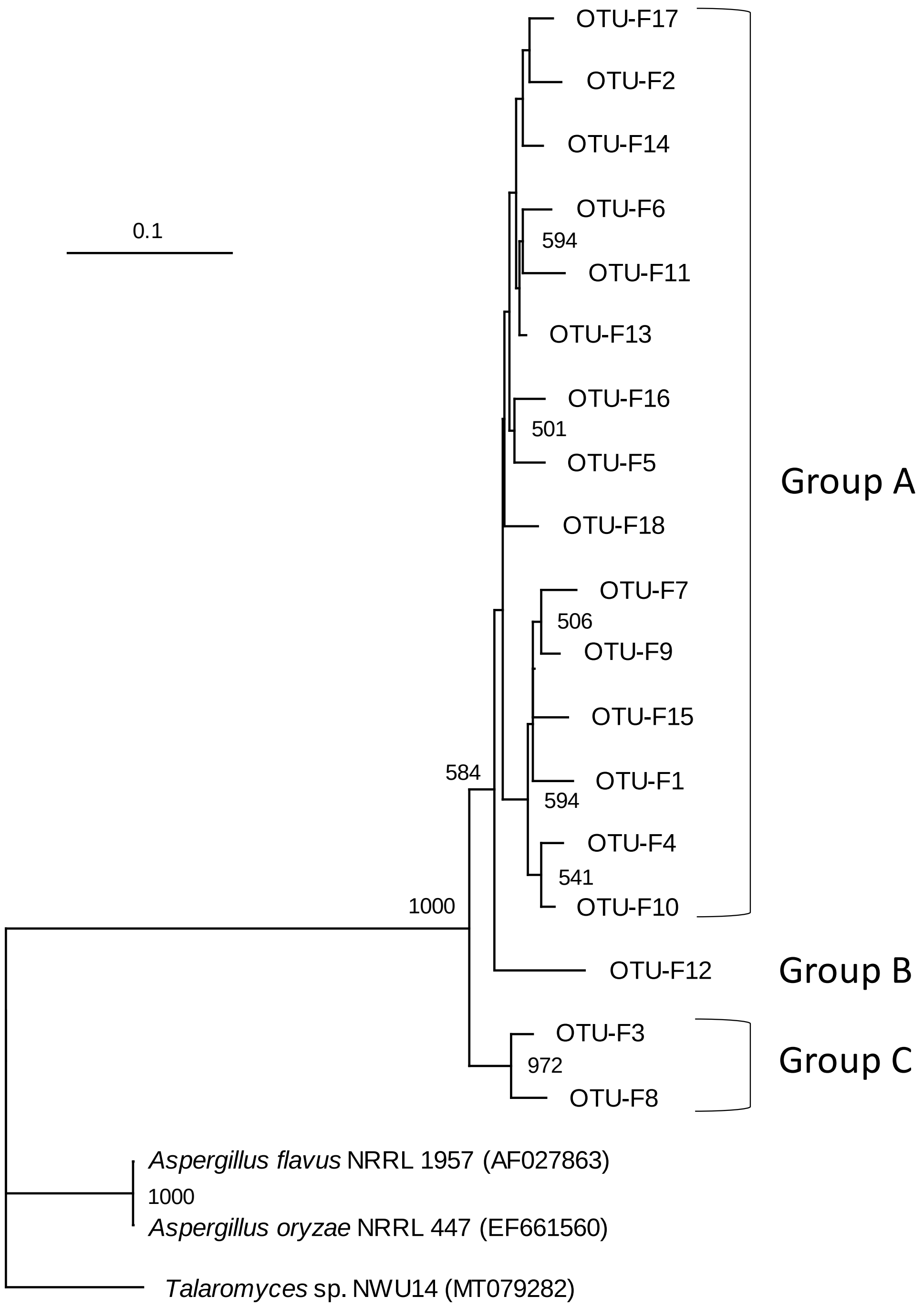

Fig. S2. Phylogenic tree analysis of representative sequences for 18 OTUs belonging to *Bipolaris* in Table 3. The tree was constructed using the neighbor-joining method with the sequences of the closest known species. The sequence of *Talaromyces* sp. NWU was used as an outgroup. The accession numbers for the sequences of closest known species and outgroup are given in parentheses. The numbers at the nodes are the proportions of 1000 bootstrap replicates, and values exceeding 500 are shown.

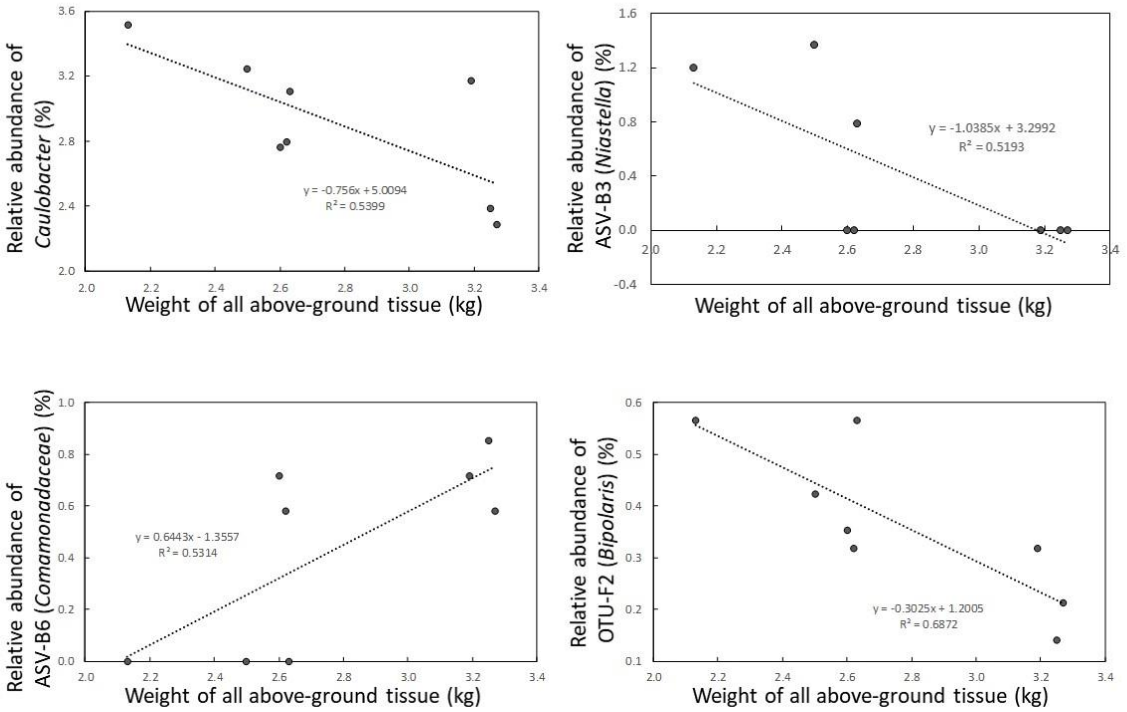

Fig. S3-A. Correlation plot of all above-ground tissue weight of napa cabbage and relative abundance of napa cabbage root-associated microbes showing statistical difference between a compound fertilizer and Chiyoda-kasei plots.

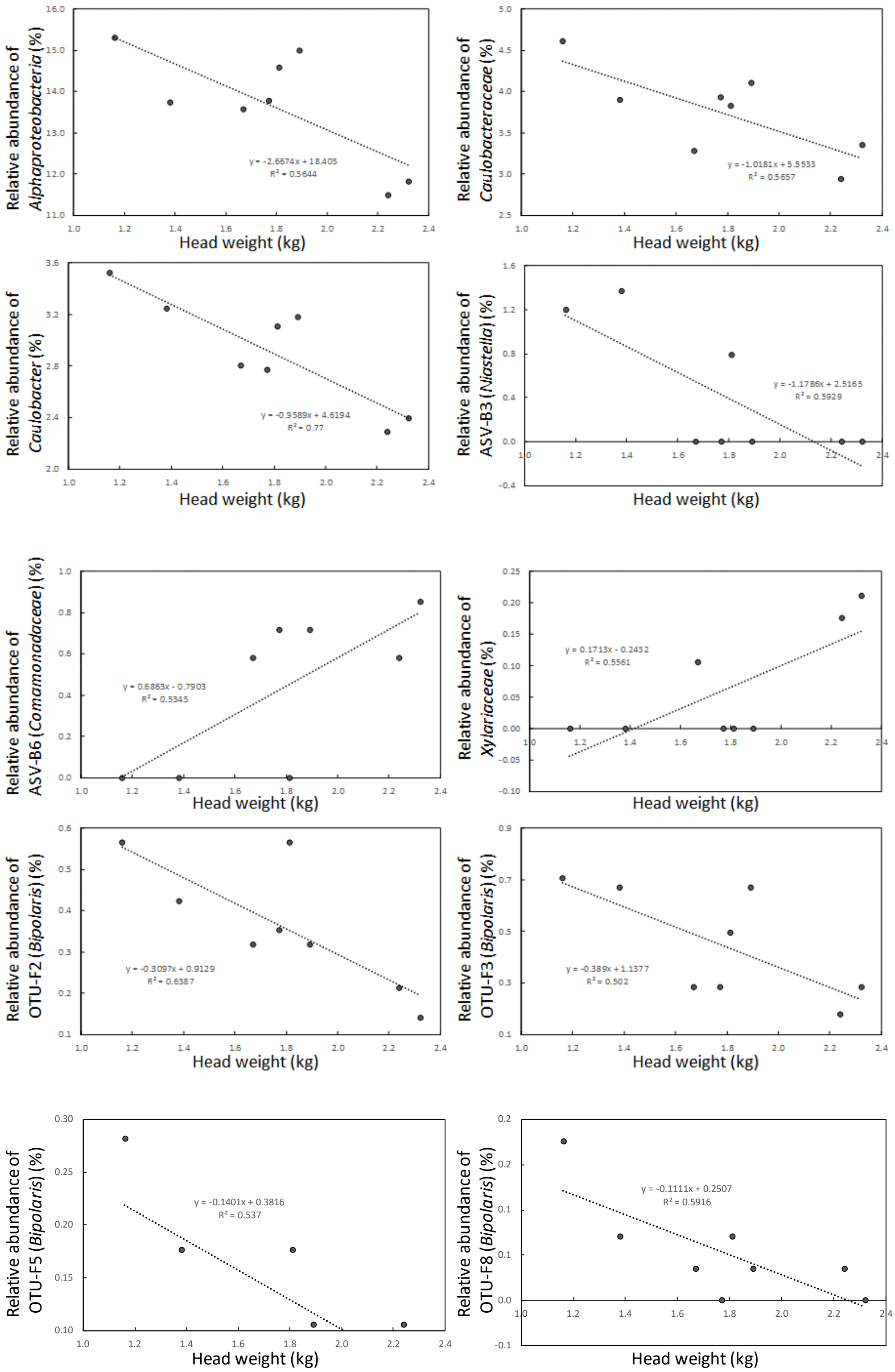

Fig. S3-B. Correlation plot of head weight of napa cabbage and relative abundance of napa cabbage root-associated microbes showing statistical difference between a compound fertilizer and Chiyoda-kasei plots.

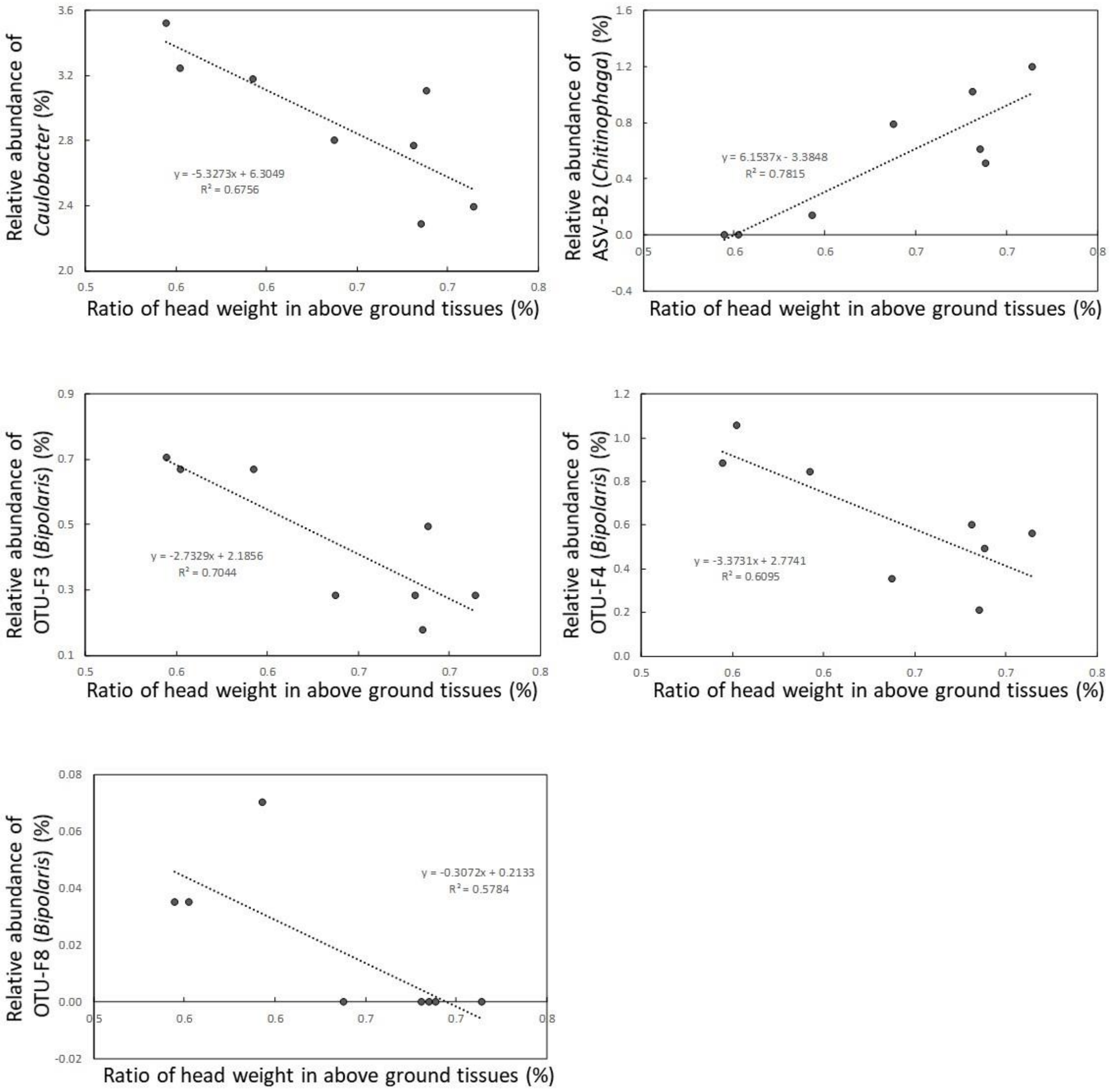

Fig. S3-C. Correlation plot of ratio of head weight in above ground tissue of napa cabbage and relative abundance of napa cabbage root-associated microbes showing statistical difference between a compound fertilizer and Chiyoda-kasei plots.
